# Effects of *ADIPOQ* polymorphisms on PCOS risk: a meta-analysis

**DOI:** 10.1186/s12958-018-0439-6

**Published:** 2018-12-03

**Authors:** Zhengling Liu, Zengyan Wang, Changhong Hao, Yonghui Tian, Jingjing Fu

**Affiliations:** Department of gynaecology, Linyi Central Hospital, No.17 Jiankang Road of Yishui county, Linyi, 276400 Shandong China

**Keywords:** Adiponectin (*ADIPOQ*), Gene polymorphisms, Polycystic ovary syndrome (PCOS), Meta-analysis

## Abstract

**Background:**

Whether adiponectin (*ADIPOQ*) polymorphisms are associated with the risk of polycystic ovary syndrome (PCOS) remain controversial. Therefore, we performed this study to better explore correlations between *ADIPOQ* polymorphisms and PCOS risk.

**Methods:**

Literature retrieve was conducted in PubMed, Medline and Embase. Odds ratios (ORs) and 95% confidence intervals (CIs) were calculated.

**Results:**

Eighteen studies were enrolled for analyses. Pooled overall analyses showed that rs1501299 polymorphism was significantly associated with PCOS risk (recessive model: *p* = 0.02, OR = 0.77, 95%CI 0.62–0.95; allele model: *p* = 0.001, OR = 1.15, 95%CI 1.06–1.26). Further subgroup analyses according to ethnicity of participants revealed that rs1501299 and rs2241766 polymorphisms were both significantly correlated with PCOS risk in Caucasians. In addition, rs1501299 polymorphism was also significantly correlated with PCOS risk in East Asians.

**Conclusions:**

Our findings indicated that rs1501299 and rs2241766 polymorphisms might serve as genetic biomarkers of PCOS in certain ethnicities.

## Background

Polycystic ovary syndrome (PCOS), featured by oligomenorrhea, polycystic ovaries, anovulatory infertility, hyperandrogenism, insulin resistance or hyperinsulinemia, and an elevated risk of multiple metabolic diseases, is an extremely common reproductive endocrine disorder, with an estimated prevalence of approximately 5–10% in women of childbearing age [1–3]. Although the exact cause of PCOS remains unclear, mounting evidence supports that genetic factors play vital roles in its pathogenesis. First, family clustering of PCOS was not uncommon, and first-degree relatives of PCOS patients suffered an increased risk of developing PCOS and its associated disorders [4, 5]. Second, various genetic variants were found to be correlated with a higher PCOS risk [6]. However, PCOS is a highly heterogeneous disorder and genetic determinants underlying PCOS are still poorly understood [7, 8].

Adiponectin (ADIPOQ), a multifunctional adipocytokine that is primarily secreted by adipocytes, plays a pivotal role in regulating energy and material metabolism [9]. Previous studies showed that expression level of adiponectin was significantly reduced in patients with various metabolic disorders such as diabetes, obesity and insulin resistance, which suggested that adipoenctin might be involved in the pathogenesis of above-mentioned diseases [10, 11]. Considering the metabolic nature of PCOS and the fact that the expression levels of adiponectin and its receptors in female reproductive organs (ovary and uterus) vary in different phases of oestrous cycle [12], it is biologically plausible that adiponectin might also be implicated in the occurrence and development of PCOS.

Adiponectin is encoded by the *ADIPOQ* gene located on chromosome 3q27 [13]. It was evident that two common functional *ADIPOQ* polymorphisms, rs1501299 and rs2241766, were correlated with altered serum concentration of adiponectin [14, 15]. As a result, these two polymorphisms were thought to be ideal genetic biomarkers of multiple metabolic disorders including PCOS. So far, several studies already investigated associations between these *ADIPOQ* polymorphisms and PCOS risk, but the results of these studies were controversial [16–33]. Therefore, we performed the present meta-analysis to better explore potential roles of *ADIPOQ* polymorphisms in PCOS.

## Methods

### Literature search and inclusion criteria

This meta-analysis was adhered to the Preferred Reporting Items for Systematic Reviews and Meta-analyses (PRISMA) guideline [34]. Potentially related literatures (published before September 2018) were retrieved from PubMed, Medline and Embase using the following searching strategy: (adiponectin OR ADIPOQ) AND (polymorphism OR variant OR mutation OR genotype OR allele) AND (polycystic ovary syndrome OR PCOS). Furthermore, the references of retrieved articles were also screened for other potentially relevant studies.

To test the research hypothesis of this meta-analysis, included studies must meet all the following criteria: (1) case-control study on correlations between *ADIPOQ* polymorphisms and PCOS risk; (2) provide genotypic and/or allelic frequency of investigated *ADIPOQ* polymorphisms in cases and controls; (3) full text in English or Chinese available. Studies were excluded if one of the following criteria was fulfilled: (1) not relevant to *ADIPOQ* polymorphisms and PCOS; (2) case reports or case series; (3) abstracts, reviews, comments, letters and conference presentations. For duplicate publications, we only included the study with the largest sample size for analyses.

### Data extraction and quality assessment

The following data were extracted from included studies: (1) the name of the first author; (2) publication time; (3) country and ethnicity; (4) sample size; and (5) genotypic distributions of *ADIPOQ* polymorphisms in cases and controls. Additionally, the probability value (*p* value) of Hardy-Weinberg equilibrium (HWE) was also calculated. When necessary, we wrote to the corresponding authors for raw data. We used the Newcastle-Ottawa scale (NOS) to assess the quality of eligible studies [35]. This scale has a score range of zero to nine, and studies with a score of more than seven were thought to be of high quality. Two reviewers conducted data extraction and quality assessment independently. Any disagreement between two reviewers was solved by discussion until a consensus was reached.

### Statistical analysis

All statistical analyses were conducted with Review Manager Version 5.3.3 (The Cochrane Collaboration, Software Update, Oxford, United Kingdom). Odds ratios (ORs) and 95% confidence intervals (CIs) were calculated to estimate strength of associations between *ADIPOQ* polymorphisms and PCOS risk in all possible genetic models, and *p* values ≤0.05 were considered to be statistically significant. Between-study heterogeneities were evaluated with I^2^ statistic. If I^2^ was greater than 50 %, random-effect models (REMs) would be used to pool the data. Otherwise, fixed-effect models (FEMs) would be employed for synthetic analyses. Subgroup analyses by ethnicity were subsequently performed. Sensitivity analyses were conducted to examine the stability of synthetic results. Funnel plots were used to evaluate possible publication bias.

## Results

### Characteristics of included studies

**Fig. 1:**
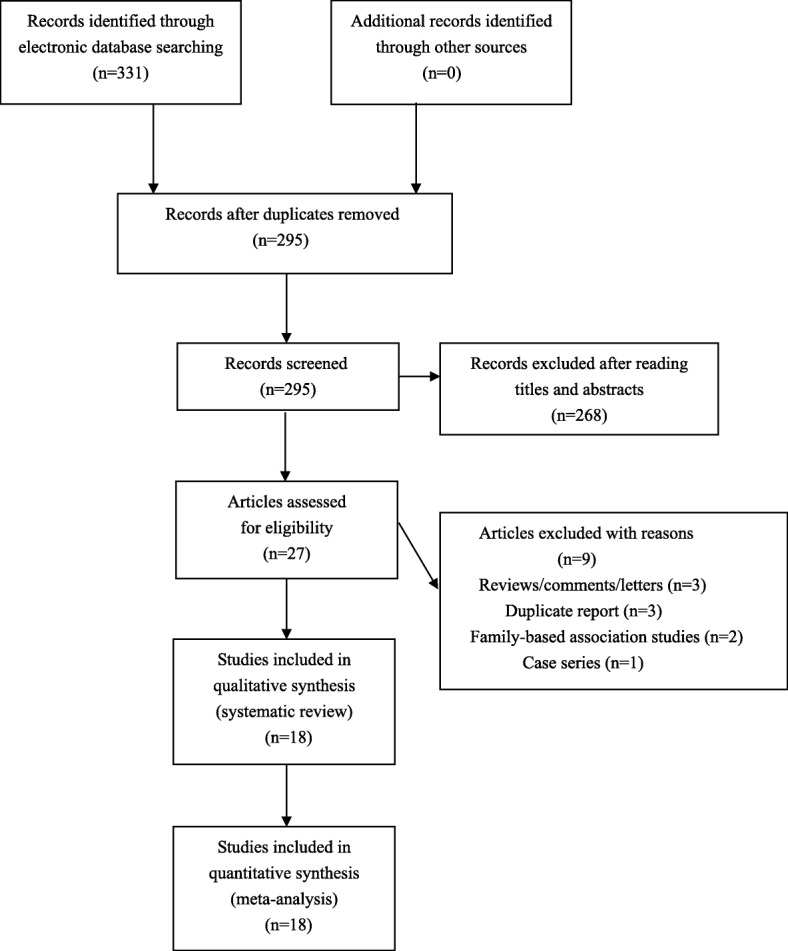
Flowchart of study selection for the present study

We found 331 potential relevant articles. Among these articles, a total of 18 eligible studies were finally included for synthetic analyses (see Fig. 1). The NOS score of eligible articles ranged from 7 to 8, which indicated that all included studies were of high quality. Baseline characteristics of included studies were shown in Table 1.

**Table 1:** The characteristics of included studies

First author, year	Country	Ethnicity	Age (years) Case/Control	Sample size	Genotype distribution		*P*-value for HWE	NOS score
					Cases Controls			
**rs1501299 G/T**					GG/GT/TT	GG/GT/TT		
Alfaqih 2018 [16]	Jordon	West Asian	23.9/24.2	154/151	62/77/15	64/54/33	0.002	8
Czeczuga-Semeniuk 2018 [17]	Poland	Caucasian	24.6/23.2	294/78	156/117/21	25/49/4	0.002	8
Escobar-Morreale 2006 [19]	Spain	Caucasian	NA	76/40	30/39/7	15/21/4	0.390	7
Heinonen 2005 [21]	Finland	Caucasian	NA	143/245	77/58/8	110/110/25	0.744	7
Li 2011 [22]	Korea	East Asian	NA	144/159	61/73/10	48/87/24	0.131	7
Nambiar 2016 [23]	India	West Asian	28.6/31.1	282/200	94/165/23	86/99/15	0.060	8
Pau 2013 [25]	USA	Mixed	NA	525/472	NA	NA	NA	7
Radavelli-Bagatini 2013 [26]	Brazil	Mixed	NA	80/1500	42/27/11	671/672/157	0.556	7
Ramezani Tehrani 2013 [27]	Iran	West Asian	26.6/30.8	186/156	92/76/18	77/71/8	0.100	7
Ranjzad 2012 [28]	Iran	West Asian	27.1/31.1	181/181	92/77/12	91/79/11	0.254	8
San Millán 2004 [29]	Spain	Caucasian	24.6/31.1	72/42	28/34/10	18/20/4	0.643	7
Xita 2005 [30]	Greece	Caucasian	23.7/24.8	100/140	39/49/12	52/73/15	0.152	7
Yoshihara 2009 [31]	Japan	East Asian	29.1/29.8	38/97	19/15/4	58/24/15	< 0.001	8
Zhang 2008 [32]	China	East Asian	28.7/29.6	120/120	56/46/18	41/50/29	0.083	8
Zhang 2015 [33]	China	East Asian	27.0/27.2	207/192	119/78/10	95/75/22	0.229	7
**rs2241766 T/G**					TT/TG/GG	TT/TG/GG		
Alfaqih 2018 [16]	Jordon	West Asian	23.9/24.2	154/149	92/48/14	93/42/14	0.008	8
Czeczuga-Semeniuk 2018 [17]	Poland	Caucasian	24.6/23.2	294/78	255/39/0	62/16/0	0.313	8
Demirci 2010 [18]	Turkey	Caucasian	24.1/23.8	96/93	70/20/6	74/16/3	0.091	8
Escobar-Morreale 2006 [19]	Spain	Caucasian	NA	76/40	55/20/1	26/13/1	0.673	7
Haap 2005 [20]	Germany	Caucasian	27.4/38.9	53/542	38/8/7	414/112/16	0.016	8
Heinonen 2005 [21]	Finland	Caucasian	NA	143/245	125/17/1	222/22/1	0.572	7
Li 2011 [22]	Korea	East Asian	NA	144/159	79/59/6	72/84/3	< 0.001	7
Nambiar 2016 [23]	India	West Asian	28.6/31.1	282/200	213/60/9	156/40/4	0.453	8
Panidis 2004 [24]	Greece	Caucasian	23.4/29.4	132/100	92/33/7	81/17/2	0.340	7
Radavelli-Bagatini 2013 [26]	Brazil	Mixed	NA	80/1500	64/14/2	1122/356/22	0.297	7
Ramezani Tehrani 2013 [27]	Iran	West Asian	26.6/30.8	186/156	142/42/2	106/46/4	0.707	7
Ranjzad 2012 [28]	Iran	West Asian	27.1/31.1	181/181	144/34/2	121/54/6	0.993	8
San Millán 2004 [29]	Spain	Caucasian	24.6/31.1	72/42	48/22/2	29/12/1	0.853	7
Xita 2005 [30]	Greece	Caucasian	23.7/24.8	100/140	77/23/0	106/30/4	0.306	7
Yoshihara 2009 [31]	Japan	East Asian	29.1/29.8	38/97	19/19/0	53/29/15	0.004	8
Zhang 2008 [32]	China	East Asian	28.7/29.6	120/120	57/54/9	74/42/4	0.504	8
Zhang 2015 [33]	China	East Asian	27.0/27.2	207/192	106/84/17	98/75/19	0.409	7

*PCOS* Polycystic ovary syndrome, *HWE* Hardy-Weinberg equilibrium, *NOS* Newcastle-Ottawa scale, *NA* Not available

### Overall and subgroup analyses

To investigate potential correlations between *ADIPOQ* polymorphisms and PCOS risk, fifteen studies about rs1501299 polymorphism and seventeen studies about rs2241766 polymorphism were included for pooled analyses. A significant association with PCOS risk was detected for rs1501299 (recessive model: *p* = 0.02, OR = 0.77, 95%CI 0.62–0.95; allele model: *p* = 0.001, OR = 1.15, 95%CI 1.06–1.26) polymorphism in overall analyses. Further subgroup analyses according to ethnicity of participants revealed that rs1501299 and rs2241766 polymorphisms were both significantly correlated with PCOS risk in Caucasians. In addition, rs1501299 polymorphism was also significantly correlated with PCOS risk in East Asians (see Table 2).

**Table 2:** Results of overall and subgroup analyses for *ADIPOQ* gene polymorphisms and PCOS

Population	Sample size	Dominant comparison			Recessive comparison			Additive comparison			Allele comparison		
		*P* value	OR (95%CI)	I^2^ statistic	*P* value	OR (95%CI)	I^2^ statistic	*P* value	OR (95%CI)	I^2^ statistic	*P* value	OR (95%CI)	I^2^ statistic
**rs1501299 G/T**		GG vs. GT + TT			TT vs. GG + GT			GT vs. GG + TT			G vs. T		
Overall	2602/3773	0.10	1.17 (0.97–1.42)	52%	**0.02**	**0.77 (0.62–0.95)**	46%	0.52	0.94 (0.76–1.15)	58%	**0.001**	**1.15 (1.06–1.26)**	43%
Caucasian	685/545	**0.007**	**1.40 (1.09–1.79)**	42%	0.79	0.94 (0.62–1.45)	0%	**0.01**	**0.73 (0.58–0.93)**	48%	**0.03**	**1.22 (1.02–1.46)**	22%
East Asian	509/568	**0.006**	**1.41 (1.10–1.81)**	37%	**0.0003**	**0.48 (0.32–0.71)**	0%	0.76	0.96 (0.75–1.23)	17%	**0.0001**	**1.44 (1.20–1.73)**	2%
West Asian	803/688	0.19	0.87 (0.71–1.07)	4%	0.88	0.95 (0.47–1.89)	70%	0.30	1.19 (0.85–1.68)	62%	0.52	0.95 (0.81–1.11)	31%
**rs2241766 T/G**		TT vs. TG + GG			GG vs. TT + TG			TG vs. TT + GG			T vs. G		
Overall	2358/4034	0.63	1.03 (0.91–1.18)	46%	0.54	1.10 (0.81–1.50)	41%	0.44	0.95 (0.83–1.09)	44%	1.00	1.00 (0.84–1.19)	54%
Caucasian	966/1280	0.32	0.89 (0.70–1.12)	22%	**0.04**	**1.93 (1.05–3.56)**	26%	0.84	1.03 (0.80–1.31)	6%	0.11	0.84 (0.68–1.04)	46%
East Asian	509/568	0.74	0.93 (0.62–1.40)	61%	0.86	1.10 (0.40–3.02)	59%	0.54	1.17 (0.71–1.93)	73%	0.95	0.99 (0.73–1.35)	58%
West Asian	803/686	0.31	1.22 (0.83–1.80)	65%	0.56	0.85 (0.49–1.47)	8%	0.31	0.83 (0.59–1.18)	53%	0.32	1.19 (0.84–1.69)	67%

*OR* Odds ratio, *CI* Confidence interval, *NA* Not available. *PCOS* Polycystic ovary syndrome

The values in bold represent there is statistically significant differences between cases and controls

### Sensitivity analyses

We performed sensitivity analyses by excluding studies that deviated from HWE. No alterations of results were detected in sensitivity analyses, which suggested that our findings were statistically reliable.

### Publication biases

Publication biases were evaluated with funnel plots. We did not find obvious asymmetry of funnel plots in any comparisons, which indicated that our findings were unlikely to be impacted by severe publication biases.

## Discussion

To the best of our knowledge, this is so far the most comprehensive meta-analysis on correlations between *ADIPOQ* polymorphisms and PCOS risk. Our overall and subgroup analyses demonstrated that rs1501299 and rs2241766 polymorphisms were both significantly correlated with PCOS risk in Caucasians. Moreover, rs1501299 polymorphism was also significantly correlated with PCOS risk in East Asians.

There are several points that need to be addressed about this meta-analysis. Firstly, previous experimental studies showed that mutant alleles of investigated polymorphisms were correlated with decreased adiponectin generation, which may partially explain our positive findings [14, 15]. Secondly, the pathogenic mechanism of PCOS is highly complex, and hence it is unlikely that a single gene polymorphism could significantly contribute to its development. As a result, to better illustrate potential correlations of certain gene polymorphisms with PCOS, we strongly recommend further studies to perform haplotype analyses and explore potential gene-gene interactions.

As with all meta-analysis, this study certainly has some limitations. First, our results were derived from unadjusted analyses due to lack of raw data, and lack of further adjusted analyses for potential confounding factors may impact the reliability of our findings [36]. Second, obvious heterogeneities were found in several subgroups, which indicated that the controversial results of included studies could not be fully explained by differences in ethnic background, and other baseline characteristics of participants may also contribute to between-study heterogeneities [37]. Third, associations between *ADIPOQ* polymorphisms and PCOS risk may also be modified by gene-gene and gene-environmental interactions. However, most eligible studies ignore these potential interactions, which impeded us to perform relevant analyses accordingly [38]. To sum up, our findings should be cautiously interpreted on account of above mentioned limitations.

## Conclusions

In conclusion, our meta-analysis suggested that rs1501299 and rs2241766 polymorphisms might serve as genetic biomarkers of PCOS in certain ethnicities. However, further well-designed studies are still warranted to confirm our findings.

## Abbreviations

ADIPOQ: Adiponectin
HWE: Hardy-Weinberg equilibrium
NOS: Newcastle-Ottawa scale
PCOS: Polycystic ovary syndrome
